# Two new species of *Brachytrycherus* Arrow, 1920 from China (Coleoptera, Endomychidae)

**DOI:** 10.3897/zookeys.595.7569

**Published:** 2016-06-02

**Authors:** Ling-Xiao Chang, Wen-Xuan Bi, Guo-Dong Ren

**Affiliations:** 1 College of Life Sciences, Hebei University, Baodin 071002, China; 2 Room 401, No. 2, Lane 155, Lianhua South Road, Shanghai, 201100 China

**Keywords:** Coleoptera, Endomychidae, new species, taxonomy, China

## Abstract

Two new species of *Brachytrycherus* from China, *Brachytrycherus conaensis* **sp. n.** and *Brachytrycherus curviantennae* **sp. n.** are described and illustrated. *Brachytrycherus conaensis* **sp. n.** is the first species of the Handsome Fungus Beetles recorded feeding on Ascomycetes. A key to the species of *Brachytrycherus* known in China is provided.

## Introduction

The genus *Brachytrycherus* was established by Arrow (1920) with *Brachytrycherus perotteti* as the type species. It is a member of the largest endomychid subfamily Lycoperdininae, the monophyly of which was confirmed by the phylogenetic studies of Tomaszewska (2000, 2005).

In 2015, a large-scale phylogenetic study was presented for Cucujoidea by Robertson et al., using molecular evidence to rebuild the relationship tree of this superfamily, and established one new superfamily, Coccinelloidea Robertson et al., 2015. The Endomychidae was included in it. Through this significant study, the monophyly of subfamily Lycoperdininae is more clear, and with subfamily Epipocinae forms the sister group to Endomychinae+Stenotarsinae (Robertson et al. 2015).

Tomaszewska (2005) placed *Brachytrycherus* with another seven genera in the Amphisternus-group: *Amphisternus* Germar, 1843, *Amphistethus* Strohecker, 1964, *Cacodaemon* Thomson, 1857, *Gerstaeckerus* Tomaszewska, 2005, *Ohtaius* Chûjô, 1938, *Spathomeles* Gerstaecker, 1857 and *Stictomela* Gorham, 1886. The monophyly of this group is well supported based on the following synapomorphies: mesoventrite with intercoxal process widened laterally towards apex, overlapping parts of coxae; elytra with basal margins thickened and raised, mandible with apical tooth widely chisel-shaped, male genital segment with additional internal V- or U-shaped sclerite (Tomaszewska 2005). Since then, two new genera of the Amphisternus-group were described, *Stroheckeria* Tomaszewska, 2006 from Vietnam, and *Humerus* Chang & Ren, 2013a from China.

Strohecker (1964) in his synopsis of the tribe Amphisternini (=Amphisternus-group of Tomaszewska (2005)) listed four species of *Brachytrycherus*, (*Brachytrycherus convexus* Strohecker, 1964, *Brachytrycherus gemmatus* (Arrow, 1928), *Brachytrycherus madurensis* Arrow, 1920 and *Brachytrycherus perotetti* Arrow, 1920) of which *Brachytrycherus convexus* as a new species and *Brachytrycherus gemmatus* as a new combination moved from *Engonius*. He also provided a key to the species of *Brachytrycherus* known at that time. Prior to the present study, *Brachytrycherus* included six species (Shockley et al. 2009a): *Brachytrycherus concolor* Arrow, 1937 (Borneo), *Brachytrycherus convexus* Strohecker, 1964 (India), *Brachytrycherus femoralis* (Arrow), 1928 (Laos, Vietnam), *Brachytrycherus gemmatus* (Arrow), 1928 (Laos, Myanmar and Thailand), *Brachytrycherus madurensis* Arrow, 1920 (India, Taiwan) and *Brachytrycherus perotteti* Arrow, 1920 (India). Only one of which was previously known from China: *Brachytrycherus madurensis*.

During the examination of the Endomychidae collected in China, two new species were recognized and are described here.

## Material and methods

Type specimens of the new species described here are deposited in the following institutions or private collections:

MHBU
Museum of Heibei University, Baoding, China 


CBWX
 Collection of Wenxuan Bi, Shanghai, China 


SHEM
 Shanghai Entomology Museum, Chinese Academy of Sciences, Shanghai, China 


MIZ
Museum and Institute of Zoology, Polish Academy of Sciences, Warszawa, Poland 


The specimens were examined and described using a Nikon® SMZ800 dissecting microscope. The following measurements were made using a Leica® M205 A dissecting microscope: body length from apical margin of clypeus to apex of elytra; width across both elytra (at widest part); elytral length along suture, including scutellum. The aedeagus was boiled in 10% NaOH solution, cleaned, and finally dissected in distilled water. Habitus photos were taken using a Canon® Eos 5D III SLR camera and Canon® MP-E 65mm macro lens. All photographs were modified in Adobe Photoshop® CC 2015.

## Taxonomy

### Brachytrycherus

Taxon classificationAnimaliaColeopteraEndomychidae

Arrow, 1920

Brachytrycherus Arrow, 1920: 12.

#### Type species.

*Brachytrycherus perotteti* Arrow, 1920.

#### Diagnosis.

The species of *Brachytrycherus* resemble those of *Ohtaius* and *Gerstaeckerus* in having the body black or blackish-brown, elytral maculae transverse, most often orange or yellow. These genera share the feature of having the mandibles chisel-shaped apically. However, *Brachytrycherus* can be distinguished from these other genera by the following combination of characters: 1) body less elongate; 2) head with well-developed gular sutures; 3) mesoventral process with sides parallel; 4) maxillary laciniae with tuft of S-like setae apically (Tomaszewska 2005).

### Brachytrycherus conaensis sp. n.

Taxon classificationAnimaliaColeopteraEndomychidae

http://zoobank.org/278F3113-648B-4DFF-BBFB-D2AB2B47177A

Figs 1–2 , 5

#### Type material.

Holotype, male, Xizang, Cona, Lexiang, 2500-2600 m, 20-30.VI.2013, Wen-Xuan Bi leg. (MHBU); Paratypes, 1 female, same data as holotype. 2 females, Xizang, Medog, Beibeng, Gelincun, 1700 m, 3.VIII.2014, Wen-Xuan Bi leg. (CBWX); 3 males, 7 females, Xizang, Cuona, Lexiang, 2500 m, 6.VIII.2010, Wen-Xuan Bi leg. (CBWX); 5 males, 6 females, ditto except 15.VII.2011 (CBWX); 26 males, 11 females, ditto except 29–30.VI.2013 (CBWX); 1 male, 1 female, ditto except (MZPW); 18 males, 1 female, ditto except 2500-2600 m, 20-30.VI.2013 (CBWX); 1 female, ditto except 2700 m, 18.VI.2013 (CBWX).

#### Etymology.

The specific name is derived from the type locality.

#### Diagnosis.

*Brachytrycherus conaensis* is similar to *Brachytrycherus madurensis* in appearance, but can be differentiated by each elytron with three maculae, anterior two maculae nearly rhomboid in shape, sometimes connected to each other, and the anterior and posterior elytral maculae without dentition.

#### Description.

Length 8.2–8.3 mm. Body oval, about 1.8–1.9 times as long as wide; rather convex; shiny. Colour black with three red maculae on elytra.

Head. Antenna 11-segmented, long and rather slender, nearly 1/2 body length, with antennomeres 1–8 distinctly longer than wide; scape approximately 4.5 times as long as pedicel; antennomere 3 slightly shorter than 4 and 5 combined; antennomeres 4 nearly as long as 5, antennomeres 5–8 gradually shorter; club composed of 3 antennomeres, moderately broad, flat, loose. Maxilla with terminal palpomere elongated, almost 2.0 times as long as palpomere 3, tapering anteriorly, truncate apically.

Thorax. Pronotum 2.0–2.3 mm long, 3.2–3.3 mm wide; widest near 1/2 of pronotal length; coarsely and densely punctate; lateral margins rather narrowly bordered, sides nearly parallel; front angles produced anteriorly, rather acute; disc weakly convex, two small round raised area laterally; transverse wrinkle laterally; median furrow absent; lateral sulci linear, deep, extending to basal 1/3 length of pronotum; basal sulcus nearly straight, deep. Prosternal process rather narrowly separates procoxae; not extending beyond coxae; sides in male weakly curved outwardly, rounded apically; in female sides nearly straight, weakly truncate apically. Mesoventral process transverse, lateral margins widening apically and overlapping part of mesocoxae; posterior margin nearly straight. Elytra 5.9–6.1 mm long, 4.5–4.7 mm wide; 2.7–3.0 times as long as pronotum and 1.4 times as wide as pronotum, sides curved, widest near 1/2 length of elytron; densely and coarsely punctate; humeri rather prominent. Each elytron with three irregular red maculae. Anterior 2 elytral elytral maculae nearly rhombus, located near apical 1/4, medial macula larger than lateral one, sometimes narrowly connected. Posterior macula transverse, anterior margin shallowly emarginate or nearly straight, posterior margin U-shaped, widely emarginate.. All tibiae with sexual characters; protibiae in male with concavity on inner edge of apical 1/4, in female without concavity; mesotibiae abruptly curved from near apical 1/3 to apex, in female gently curved; metatibiae in male abruptly widened from near 1/3 length to apex, in female gently widened.

Abdomen with five ventrites in both sexes. Ventrite 5 with lateral margins gently converging posteriorly, three pairs of longitudinal short wrinkles laterally; posterior margin weakly curved medially in male; in female ventrite 5 lateral margins abruptly converging posteriorly, without longitudinal wrinkles; posterior margin truncate, nearly straight medially. Aedeagus (Fig. 5) rather long, heavily sclerotized, straight. Median lobe branched apically; branch long and rather straight, abruptly raised near basal 1/3 length, gently converging apically, flat, acute and weakly reflexed apically. Tegmen basal, comparatively large, ring-shaped.

**Figures 1–2.**
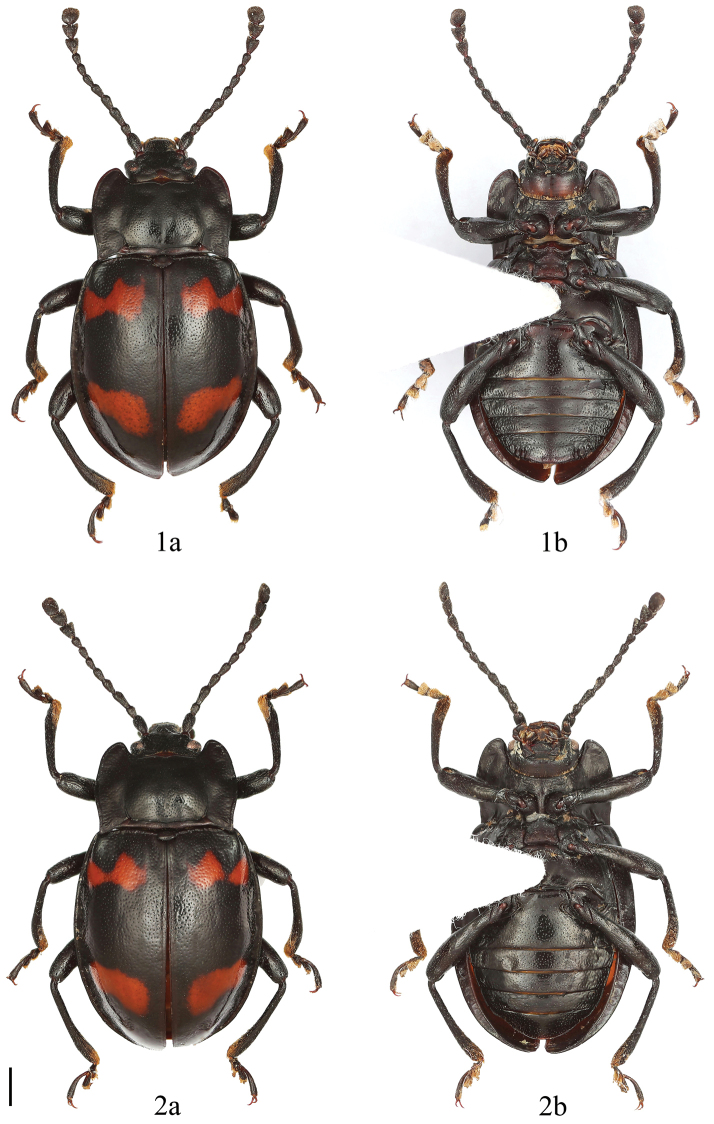
Dorsal and ventral habitus of *Brachytrycherus conaensis* sp. n. **1** male **2** female. a = dorsal view, b = ventral view. Scale bar 1 mm.

**Figures 3–4.**
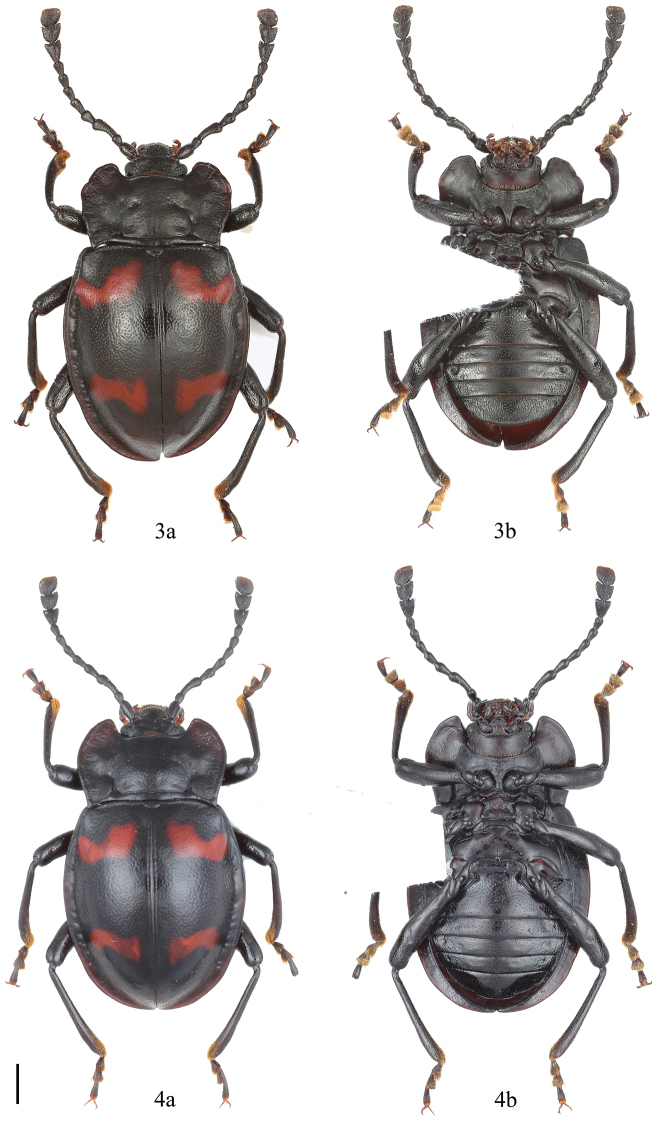
Dorsal and ventral habitus of *Brachytrycherus curviantennae* sp. n. **3** male **4** female. **a** = dorsal view, **b** = ventral view. Scale bar 1 mm.

**Figures 5–6.**
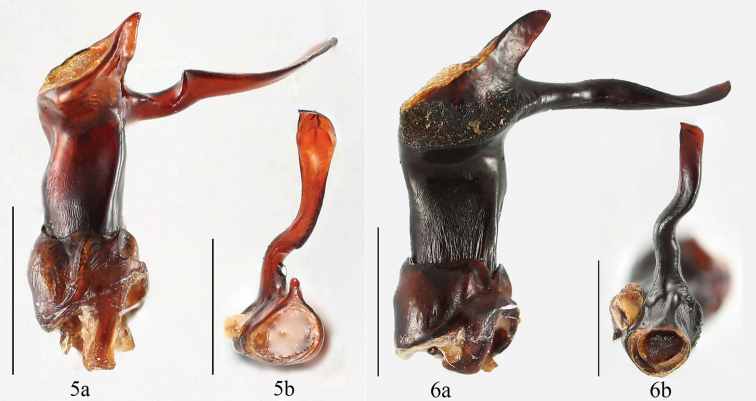
Aedeagus **5** *Brachytrycherus conaensis* sp. n.; **6** *Brachytrycherus curviantennae* sp. n. **a** = lateral view, **b** = apical view. Scale bars 1 mm.

#### Biology and ecology.

Almost all individuals were found active on fence, woodpile or timber piles within the village and its surrounding area at night (Figs 9–11). Some larvae and adults were found (sometimes at the same time) feeding on the surface of the perithecia or spores of *Daldinia concentrica* (Xylariaceae) (Fig. 10), seeming to prefer the asexual phase; however, individuals were also found on mature ascocarps.

**Figures 7–11.**
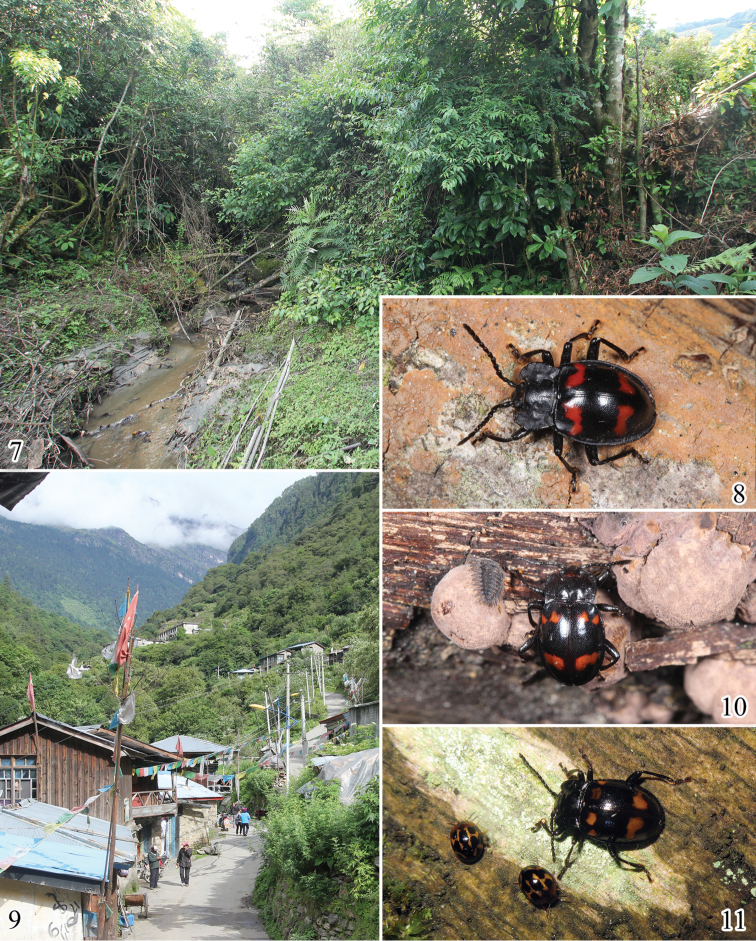
Habitats of *Brachytrycherus* species. **7** large clump of Fagaceae plants of collecting site in Xizang, China **8** male of *Brachytrycherus curviantennae* sp. n. (arranged) **9** village of collecting site in Xizang, China **10** male of *Brachytrycherus conaensis* sp. n. and larva on the wood pile **11** female of *Brachytrycherus conaensis* sp. n. feeding on the lichen growing on wood.

Based on the study of the natural history of the handsome fungus beetles (Shockley et al. 2009b), this report is possibly the first record of the handsome fungus beetles feeding on ascomycetes. In addition, some individuals were found active on the wood without fungus, and may be feeding on lichen growing on the wood (Fig. 11). This species association may not be host-specific.

### Brachytrycherus curviantennae sp. n.

Taxon classificationAnimaliaColeopteraEndomychidae

http://zoobank.org/CA118D1D-C4CD-4DB6-BEFB-22D840A76F1A

Figs 3–4 , 6

#### Type material.

Holotype, male, Xizang, Medog, 1500 m, 20.VIII.2013, Wen-Xuan Bi leg. (SHEM); Paratypes, 1 female, Xizang, Medgo, Beibeng, Gelincun, 3.VIII.2014, Wen-Xuan Bi leg. (MHBU); 1 female, Xizang, Medgo, Beibeng, Gelincun, 3.VIII.2014, Wen-Xuan Bi leg. (CBWX).

#### Etymology.

The name refers to the antennomere 3 distinctly outwardly curved.

#### Diagnosis.

*Brachytrycherus curviantennae* is similar to *Brachytrycherus madurensis* in appearance, but can be differentiate by antennomere 3 distinctly curved outwards, pronotum sides strongly curved, elytral maculae with front and hind margins emarginate.

#### Description.

Length 8.5–9.4 mm. Body broadly oval, approximately 1.6–1.8 times as long as wide; convex; shiny. Colour black with four red maculae on elytra.

Head. Antenna 11-segmented, long and rather slender, nearly 1/2 body length, with antennomeres 1–8 distinctly longer than wide; scape approximately 4.5 times as long as pedicel; antennomere 3 distinctly curved, and nearly as long as 4 and 5 combined; antennomere 4 as long as 5, antennomeres 5–8 gradually shorter; club composed of 3 antennomeres, broad and flat. Maxilla with terminal palpomere elongate, almost 2.0 times as long as palpomere 3, tapering anteriorly, truncate apically.

Thorax. Pronotum 2.0–2.4 mm long, 4.1–4.2 mm wide; widest near 1/2 of pronotal length; coarsely and densely punctate; lateral margins narrowly bordered, sides in male wavy and strongly curved; in female sides smooth and strongly curved,; front angles produced anteriorly, blunt; disc weakly convex, with two large round raised areas laterally; transverse wrinkle laterally; median furrow absent; lateral sulci linear, deep, extending to basal 1/3 of pronotal length; basal sulcus nearly straight, deep. Prosternal process moderately separates the procoxae; sides weakly curved outwardly , weakly truncate apically. Mesoventral process transverse, lateral margins widening apically and overlapping part of mesocoxae; posterior margin nearly straight. Elytra 6.0–7.1 mm long, 5.1–5.2 mm wide; 3.0 times as long as pronotum and 1.2–1.3 times as wide as pronotum, sides curved, widest near 1/2 length of elytron; densely and coarsely punctate; humeri rather prominent. Each elytron with two transverse, irregular in shape red maculae. Anterior elytral macula nearly cymbiform, anterior margin widely U-shaped and deeply emarginate, posterior margin weakly wavy. Posterior macula transverse, inversely cymbiform, anterior margin weakly wavy, posterior margin widely U-shaped and deeply emarginate. Protibiae in male with concavity on inner edge of apical 1/3, in female without concavity; mesotibiae abruptly curved from near apical 1/3 to apex, in female gently curved; metatibiae in male abruptly widened from near 1/3 length to apical 1/4, in female gently widened.

Abdomen with 5 ventrites in both sexes. Ventrite 5 with lateral margins gently converging posteriorly, posterior margin widely rounded medially in male; in female ventrite 5 lateral margins abruptly converging posteriorly, posterior margin truncate, nearly straight medially. Aedeagus (Figs 6) rather long, heavily sclerotized, straight. Median lobe branched apically; branch long and rather straight, gently rising from about basal 1/3 to apical 1/3, flat, acute and weakly reflexed apically. Tegmen placed basally, comparatively large, ring-shaped.

#### Biology and ecology.

The male was hand collected by simple searching, as it is active on branches at night (Fig. 7). Two females were collected by shaking the tree from a large clump of dead wood of Fagaceae plants (Fig. 8).

### Key to the species of *Brachytrycherus* known in China

**Table:** 

1	Antennomere 3 distinctly outwardly curved; pronotum sides strongly curved	***Brachytrycherus curviantennae* sp. n.**
–	Antennomere 3 straight; sides of pronotum nearly parallel	**2**
2	Each elytron bearing 2 transverse, strongly dentate maculae	***Brachytrycherus madurensis***
–	Each elytron bearing 3 maculae, anterior 2 maculae nearly rhomboid, sometimes connected to each other; elytral maculae not dentate	***Brachytrycherus conaensis* sp. n.**

## Conclusions

Prior to this study, only *Brachytrycherus madurensis* was recorded from China (Taiwan) (Shockley et al. 2009a). Two new species comprise the first record of *Brachytrycherus* from mainland China.

## Supplementary Material

XML Treatment for Brachytrycherus

XML Treatment for Brachytrycherus conaensis

XML Treatment for Brachytrycherus curviantennae
