# Corrigenda: Two new species of *Brachytrycherus* Arrow, 1920 from China (Coleoptera, Endomychidae). ZooKeys 595: 137–146. doi: 10.3897/zookeys.595.7569

**DOI:** 10.3897/zookeys.638.10994

**Published:** 2016-12-08

**Authors:** Ling-Xiao Chang, Wen-Xuan Bi, Guo-Dong Ren

**Affiliations:** 1 College of Life Sciences, Hebei University, Baodin 071002, China; 2 Room 401, No. 2, Lane 155, Lianhua South Road, Shanghai, 201100 China

When describing the new species *Brachytrycherus conaensis* sp. n., the information of two female paratypes is incorrect and should be deleted. When describing another the new species, *Brachytrycherus curviantennae* sp. n., the information of one female paratype is incomplete and should be added “1700 m” altitude; the information of another female paratype should be reduced to “ditto except (CBWX)”, and in all the information of paratype, the word “Medgo” is a spelling error, and the correct spelling is “Medog”.

The correct information *Brachytrycherus conaensis* sp. n. and in *Brachytrycherus curviantennae* sp. n. should be as follows:

Paratypes, 1 female, same data as holotype. 3 males, 7 females, Xizang, Cuona, Lexiang, 2500 m, 6.VIII.2010, Wen-Xuan Bi leg. (CBWX); 5 males, 6 females, ditto except 15.VII.2011 (CBWX); 26 males, 11 females, ditto except 29–30.VI.2013 (CBWX); 1 male, 1 female, ditto except (MZPW); 18 males, 1 female, ditto except 2500–2600 m, 20–30.VI.2013 (CBWX); 1 female, ditto except 2700 m, 18.VI.2013 (CBWX).

Paratypes, 1 female, Xizang, Medog, Beibeng, Gelincun, 1700 m, 3.VIII.2014, Wen-Xuan Bi leg. (MHBU); 1 female, ditto except (CBWX).

